# Intraventricular pressure gradient: A novel tool to assess the post-infarction chronic congestive heart failure

**DOI:** 10.3389/fcvm.2022.944171

**Published:** 2022-08-16

**Authors:** Hussein M. El-Husseiny, Eman A. Mady, Danfu Ma, Lina Hamabe, Ken Takahashi, Ryou Tanaka

**Affiliations:** ^1^Laboratory of Veterinary Surgery, Department of Veterinary Medicine, Faculty of Agriculture, Tokyo University of Agriculture and Technology, Fuchu-shi, Japan; ^2^Department of Surgery, Anesthesiology, and Radiology, Faculty of Veterinary Medicine, Benha University, Toukh, Egypt; ^3^Laboratory of Veterinary Physiology, Department of Veterinary Medicine, Faculty of Agriculture, Tokyo University of Agriculture and Technology, Fuchu-shi, Japan; ^4^Department of Animal Hygiene, Behavior and Management, Faculty of Veterinary Medicine, Benha University, Toukh, Egypt; ^5^College of Veterinary Medicine, Nanjing Agricultural University, Nanjing, China; ^6^Department of Pediatrics and Adolescent Medicine, Juntendo University Graduate School of Medicine, Bunkyo, Japan

**Keywords:** intraventricular pressure gradient, IVPG, congestive heart failure, myocardial infarction, diastolic dysfunction, echocardiography

## Abstract

Congestive heart failure (CHF), the leading cause of death, is deemed a grave sequel of myocardial infarction (MI). The employment of left ventricular end-diastolic pressure (LVEDP), as a primary indication of CHF, becomes restricted owing to the potential impairment of heart function and caused injury to the aortic valve during its measurement. Echocardiography is the standard technique to detect cardiac dysfunction. However, it exhibits a low capacity to predict the progression of CHF post chronic MI. Being extremely sensitive, noninvasive, and preload-independent, intraventricular pressure gradient (IVPG) was lately introduced to evaluate cardiac function, specifically during cardiomyopathy. Yet, the utility of its use to assess the CHF progression after chronic MI was not investigated. Herein, in the current research, we aimed to study the efficacy of a novel echocardiographic-derived index as IVPG in the assessment of cardiac function in a chronic MI rat model with CHF. Fifty healthy male rats were involved, and MI was surgically induced in 35 of them. Six months post-surgery, all animals were examined using transthoracic conventional and color M-mode echocardiography (CMME) for IVPG. Animals were euthanized the following day after hemodynamics recording. Gross pathological and histological evaluations were performed. J-tree cluster analysis was conducted relying on ten echocardiographic parameters suggestive of CHF. Animals were merged into two main clusters: CHF+ (MI/HF + group, *n* = 22) and CHF– (*n* = 28) that was joined from Sham (*n* = 15), and MI/HF– (*n* = 13) groups. MI/HF+ group showed the most severe echocardiographic, hemodynamic, anatomic, and histologic alterations. There was no significant change in the total IVPG among various groups. However, the basal IVPG was significantly increased in MI/HF+ group compared to the other groups. The remaining IVPG measures were considerably increased in the MI/HF+ group than in the Sham one. The segmental IVPG measures were significantly correlated with the anatomical, histological, echocardiographic, and hemodynamic findings except for the heart rate. Moreover, they were significant predictors of CHF following a long-standing MI. Conclusively, IVPG obtained from CMME is a substantially promising noninvasive tool with a high ability to detect and predict the progression of CHF following chronic MI compared to conventional echocardiography.

## Introduction

Congestive heart failure (CHF), the chief cause of death, is a frequent life-threatening outcome of myocardial infarction (MI). The progression of heart failure may be attributed either to systolic dysfunction with deficient blood ejection during systole or diastolic dysfunction with imperfect filling during diastole (1). Several heart failure cases present meaningful systolic dysfunction and are categorized as patients with heart failure with reduced ejection fraction (HFrEF) (2). Regional heterogenic left ventricle (LV) contraction is the chief cause of systolic dysfunction in HFrEF. Meanwhile, in patients with MI, it is more obvious near the fibrotic scar (3). Being practical and relatively cost-effective, rats represent the most widely used animal model to study MI and CHF (4, 5). The ligation of the coronary artery or the high site ligation of its branch (left anterior descending [LAD]) results in massive cardiac dysfunction, LV remodeling, and different size MI. However, progression of CHF following MI occurs only in animals with large size infarctions. Moreover, the size of MI is an important factor in defining the time utilized for this transition (5).

The detection of CHF in patients with MI is essential to evaluate the efficacy of treatment. It usually depends on physical, clinical, and echocardiographic evidence (6). In rats, the diagnosis of CHF depending on the clinical findings is subjective with high experience required. Besides, some common clinical features of CHF, as body weight (BW) decrement, are easily disrupted by other indicatives such as local or systemic congestion and edema. Meanwhile, many clinical findings suggestive of CHF can be precisely assessed only following euthanasia (7). Hence, left ventricular end-diastolic pressure (LVEDP) was the chief tool to diagnose CHF (5, 8). However, the invasive procedures of this technique with consequent disruption of the cardiac function or the possible damage of the aortic valves retain its use somewhat limited (4). Echocardiography is the gold standard method for diagnosing myocardial dysfunction in animals and humans. However, it presents a limited potential to predict the progression of CHF following chronic MI. With the rapid advancement of echocardiographic technology and the establishment of color M-mode echocardiography (CMME), intraventricular pressure difference (IVPD) has started to be used as a noninvasive marker for heart function evaluation. An index that focuses on intraventricular blood flow and proved to be a sensitive indicator of the diastolic function during early diastole (9). IVPD, which is created in the early stages of diastole when the apical pressure drops below the basal pressure, is the pressure differential between various sites within the LV (10). It also has the benefit of repeatability. Using Euler's equation, IVPD is calculated from CMME images (11). As a result, it is possible to assess the ventricular function noninvasively (9). This enables the IVPD between any two points in the LV to be calculated, allowing for the division of the LV into several parts for in-depth analysis. The intraventricular pressure gradient (IVPG) was presented as a reliable, noninvasive, preload-independent, and highly precise diastolic function index to evaluate the cardiac structure and function, chiefly during myocardiopathy. It is calculated by dividing the IVPD by the LV length. Similar to IVPD, the IVPG also exerts the same characteristics without being influenced by LV length (11–13). The length of the LV, from the mitral valve to the apex, is typically split into four sections: the basal, mid, mid-to-apical, and apical regions. Each section has an associated IVPG index and a specific function in the diagnosis of cardiac disease. For instance, it has been noted that mid-IVPD reduces as diastolic dysfunction worsens, whereas basal-IVPD increases as congestion worsen (14, 15). Additionally, it was shown that the apical-IVPG is primarily responsible for aggressively sucking blood into the ventricle, whereas the mid-to-apical-IVPG represents the active relaxation of the LV during diastole (16, 17). In our laboratory, IVPG has been utilized to detect or predict different cardiac disease conditions in different animal models (11–13, 18, 19). Out of these studies, IVPG was proved to be a substantial noninvasive alternative with a high capacity to diagnose and even predict different cardiac affections. However, to the best of our knowledge, it has not been investigated for the evaluation of CHF following long-term MI. Hence, the main objective of the current study is to assess the feasibility of IVPG as a novel echocardiographic imaging tool to assess the progression of post-infarction chronic CHF.

## Materials and methods

### Study design

**Figure 1:**
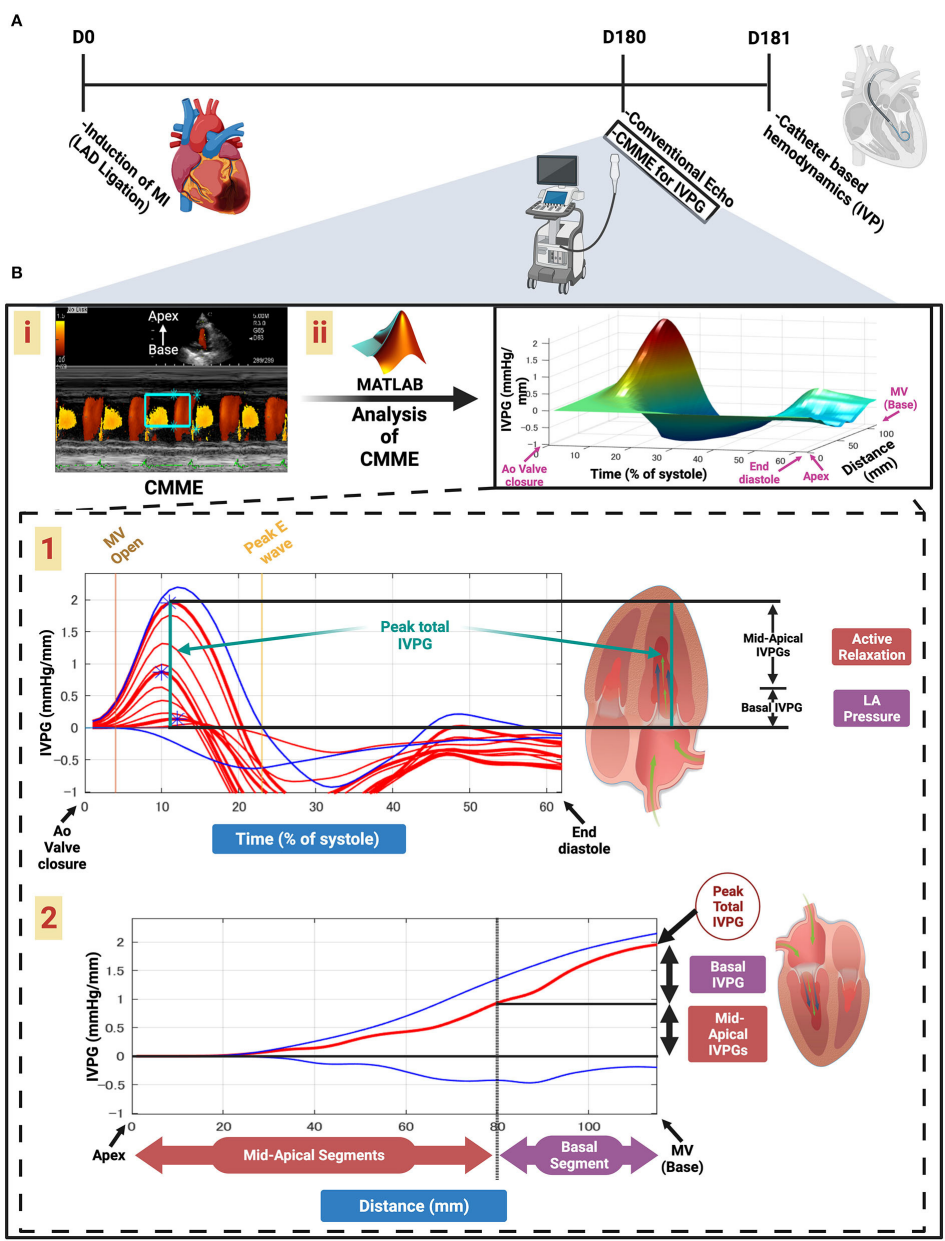
Schematic illustration of this study design and calculation of IVPG. Timeline of the study **(A)**. Color M-mode echocardiography (CMME) for intraventricular pressure gradient (IVPG) **(B)**. Apical four-chamber view CMME to trace the inflow tract from the left atrium (LA) to the apex of the left ventricle (LV) with the cursor parallel to the mitral inflow (i). CMME analysis using MATLAB software and generation of the 3D temporal and spatial profiles of the IVPG using Euler's equation over a percent of time of systolic duration from the complete closure of the aortic valve to the end of diastole. The z-axis is the distance (mm) from the apex to the mitral valve (base) (ii). Two-dimensional graph of the temporal profile of the IVPG over % of systole from the aortic valve closure to the end of diastole showing the peak total IVPG and segmental IVPGs, respectively (1). Two-dimensional spatial profile of the IVPG from the apex to the base (MV) of the heart. The red line represents the spatial profile at peak total IVPG that often happens at the level of the MV. The top and the bottom blue lines represent the inertial and the convective IVPG, respectively. Calculation of the basal and mid-apical segmental IVPGs relied on the rule of LV segmentation (one-third for basal and two-thirds for mid-apical) (2).

Transthoracic conventional and CMME for IVPG were measured in Sham (*n* = 15) and MI animals (*n* = 35) 180 days post-surgery. The next day, the animals were anesthetized and ventilated on the respirator with 2.2% isoflurane, and the hemodynamic [intraventricular pressure (IVP)] indices were recorded (Figure 1A). Then, the rats were euthanized, and the heart was separated and weighed. Fragments of lung and liver were weighed before and after drying sessions (65°C for 72 h) to evaluate the wet-to-dry weight ratio (4).

### Experimental animals

A total of 50 male Sprague Dawley rats (Kitayama Labes Co Ltd., Nagano, Japan), 3 months age, 359.92 ± 35.24 gm BW were used. Animals were housed individually at 25 ± 2°C with 12-h light/dark cycles with access to food and water *ad libitum*. All procedures were performed following the Guide for the Care and Use of Laboratory Animals, as published by the US National Institute of Health (NIH), reviewed, and approved by the Institutional Animal Care and Use Committee of Tokyo University of Agriculture and Technology (Approval No. R02-112).

Ten MI animals died; six animals died within 24 h of LAD, and the other four animals died throughout the experimental period. Died animals have been excluded from the study, and we performed the other ten rats instead. There were no deaths among Sham-operated animals. Fifty rats have been used for the statistical analysis.

### Establishment of myocardial infarction model

According to a previously described method, MI was induced (20). Briefly, rats were initially anesthetized with 4% isoflurane, then intubated with a 16G intravenous catheter and ventilated with a mixture of oxygen and 1.5–2% isoflurane, using a rodent ventilator (Harvard Apparatus, Boston). A left lateral thoracotomy was performed at the level of the 4th intercostal space. The pericardium was opened, and the left atrium was retracted to facilitate ligation of the LAD artery branch of the left coronary artery (LCA). LAD was ligated at the level of the atrioventricular junction with a 6-0 prolene suture [MI (*n* = *35*)]. The successful ligation was confirmed by the paleness of the anterior wall of the LV. Following LAD ligation, the heart was replaced into the thoracic cage, the lungs were inflated with positive pressure, and the thoracotomy was closed in layers. Animals in the Sham-operated group (*n* = *15*) underwent the same surgical procedures without LAD ligation and were used as controls. Post-surgery, all animals were monitored and received one dose of buprenorphine (0.05–0.2 mg/kg I/P) within 6 h after surgery, and another dose was administered the following morning.

### Conventional echocardiography

Six months following surgery, cardiac functions have been assessed 1 day prior to euthanasia in a total of 50 animals [Sham (*n* = *15*) and MI (*n* = *35*)]. ProSound 7 ultrasonographic system equipped with a 12-MHz transducer supported with CMME and simultaneous ECG (Hitachi-Aloka Medical Ltd., Tokyo, Japan) was used. Echocardiography was conducted according to the American Society of Echocardiography (ASE) Guidelines (21) and (22).

A two-dimensional right parasternal short-axis view of the LV using M-mode was obtained at the level of the papillary muscles. All LV structures were manually measured by the same observer using the leading-edge method of the ASE (23), which has been validated for the rat MI model (24). The obtained values were the mean of at least five consecutive cardiac cycles on the M-mode tracings. The LV internal diameter during diastole (LVIDd), LV internal diameter during systole (LVIDs), LV posterior wall diameter during diastole (LVPWd), and systole (LVPWs), ejection fraction (EF%), and fractional shortening (FS%) were obtained from that view. The LV mass (LVM) index was calculated using the following formula (25):


$$\begin{array}{l} \text{LVM}\left(\text{mg}\right)\text{ = 1}.\text{04}\times [{\left(LVIDd+LVPWd+IVSd\right)}^{3}\\ \;-LVIDd^{3}]\times 0.8+0.14 \end{array}$$

Furthermore, the left atrial systolic diameter (LADs), aortic root diastolic diameter (AoD), the left atrium to aortic diameter ratio (LA/Ao), and the Doppler assessment of the pulmonary blood flow [right ventricular outflow tract (RVOT)] indices including peak velocity (PV), cardiac output (CO), and velocity time integral (VTI) were measured using the previously mentioned position but at the level of the heart base (the level of aorta and the main pulmonary artery). Meanwhile, the Doppler assessment of the aortic blood flow [LV outflow tract (LVOT)] indices comprising PV, CO, and VTI, and the trans-mitral inflow indices comprising the early (E), the late (A) velocities, and the E/A ratio using pulsed-wave (PW) Doppler echocardiography were gained through the left apical four-chamber view. The value of isovolumetric relaxation time (IVRT) was extracted from the PW of LVOT. Moreover, Tissue Doppler imaging (TDI) was gained from the same view. The LV septal and posterior walls movement were sampled by PW TDI echocardiography with a sample volume of 0.5 mm. The TDI velocity profile includes systolic (s') and diastolic velocities [early (e') and late (a')] at the point of attachment of the mitral valve to the septal and lateral walls of the LV were recorded.

E/e' was calculated by the following formula:


$$\begin{array}{l} E/e^{\prime }=\left(E/e^{\prime }lateral+E/e^{\prime }septal\right)/2 \end{array}$$

### Color M-mode echocardiography for IVPG

The calculation of IVPG indices from the CMME using Euler's equation is illustrated in Figure 1B. The CMME was used for the evaluation of IVPG. The ultrasound machine was set to a sweep speed of 300 mm/s and color baseline shift of−64 to increase the Nyquist limit for proper tracing of the CMME. Firstly, the left apical 4-chamber view was adjusted, and the blood flow tract from the left atrium toward the LV apex across the mitral valve was optimized. After that, the M-mode was initiated to trace the inflow. Color M-mode images were saved for further offline analysis using MATLAB software (The MathWorks, Natick, MA). The CMME images were analyzed using a custom code created in MATLAB using the following image processing algorithm:


$$\begin{array}{l} \left(\partial P\right)/\left(\partial s\right)=-\rho \cdot \left(\left(\partial \text{v}\right)/\left(\partial t\right)+v\cdot \left(\partial v\right)/\left(\partial s\right)\right) \end{array}$$

where *P* is the pressure, *s* is the point along with the scan line (i.e., the line along the transmitral flow where the color Doppler M-mode measurements were collected), ρ is a constant denoting the blood density, *v* is the transmitral flow velocity, and *t* is the time. De-aliasing was used to recreate the pictures. The reconstructed velocity field may be used to compute the relative pressures in the region of interest (15). The relative pressure was calculated at each location along with the scan line as the difference between that location's pressure and the mitral annulus's pressure during aortic valve closure. It was possible to determine the temporal profile of the LV apex pressure in relation to the LA pressure by computing the line integral along with the scan line. Then, the peak IVPD from the mitral valvular annulus to the LV apex was then computed using a technique that has already been verified against direct measurements made with micro manometers (9, 26), as discussed in detail in other works (9, 15, 26). All data were averaged across at least three beats, and the mean values were exploited for the final analysis. IVPG was calculated as IVPD divided by LV length (27). For software analysis of location-reliant IVPG, the LV was divided into four parts: the basal part expressed as basal-IVPG that coordinated the pressure at the mitral valve, the apical segment known as apical-IVPG at the apex of the LV, the central part named mid-IVPG, and the part between them named mid-to-apical IVPG. The total pressure was defined as total-IVPG.

### Hemodynamics and measurement of intra-ventricular pressure indices

Through the right carotid artery, a 2-Fr microtip pressure transducer catheter (SPR-407, Mikro-Cath, Millar Instruments) was inserted retrogradely into the LV to measure the IVP and its derivatives. The transducer was connected to a Millar TCB-500 transducer control unit connected with PowerLab hardware (ML880 PowerLab 16/30, AD Instruments) and LabChart Pro software (LabChart v8, AD Instruments). Heart rate (HR), LVEDP, minimal (diastolic) blood pressure (DBP), maximal (systolic) blood pressure (SBP), and positive and negative left ventricular pressure derivatives (dP/dt_max._ and dP/dt_min._), and time constant of isovolumic relaxation [Tau (τ)] were measured. Data acquisition was performed over 10–15 cardiac cycles, and values were then averaged. During the recording of data, the ventilator was temporarily stopped to avoid respiratory-induced variations.

### Gross pathological findings

Prior to euthanasia, animals' weights were recorded. Then, they were euthanized with an overdose of isoflurane. Thoracotomy was performed to excise the heart. Moreover, fragments of the lung and the liver were obtained, and their weights were recorded before (wet weights) and after (dry weights) dryness (65°C for 72 h). The wet/dry weight ratio was calculated (4).

### Determination of infarct size (histopathological analysis)

After euthanasia, the hearts were harvested, rinsed with Phosphate buffer saline (PBS), fixed in 4% buffered formalin, embedded in paraffin, and cut into 5-μm thick sections. They were deparaffinized in xylene, dehydrated in graded ethanol mixtures, and stained with hematoxylin and eosin (H&E). To investigate the cardiac structure and fibrosis, Masson's trichrome (MTC) staining was performed. Planimetry of the endocardial and epicardial circumferences was used to measure the lengths of the infarcted and viable myocardium. The size of the infarction area (%) was calculated by dividing the sum of endocardial and epicardial infarcted ventricular lengths by the sum of total (viable and infarcted myocardium) endocardial and epicardial ventricular circumferences. The measurements were acquired from midventricular slices, under the notion that the left midventricular slice shows a close linear relationship with the sum of the measurements from all heart slices (28, 29).

### Statistical analysis

Data were presented as mean ± SD. A hierarchical cluster analysis of selected echocardiographic data was performed using the weighted pair-group average linkage method and medium Euclidean distances. Ten echocardiographic variables were chosen and used in the cluster analysis: LVIDd, LVIDd/BW, LVIDs, EF, FS, LA/Ao, LA/BW, E wave, E/A, and IVRT. The cluster analysis sets up a linkage tree allowing the assignment of cases into subsets called clusters. These can be grouped using the linkage method in which a short linkage distance implies that the cases are similar, while a long linkage distance implies that cases are different (4, 5). Groups classified according to the cluster analysis were compared by Kruskal–Wallis one-way analysis of covariance and Dunnets *post-hoc* test. A comparison of the infarct size between MI/HF+ and MI/HF– groups were conducted by the Student's *t*-test. The relationship between different IVPG indices and the echocardiographic, hemodynamic, anatomic, and histologic data was assessed by Pearson's correlation coefficients and multivariate linear regression analysis. All measures have been performed by the same examiner. Intra-observer reproducibility of the IVPG indices was tested by the Bland–Altman analyses comprising the estimation of mean bias (average difference between measurements), and the 95% limits of agreement of mean bias (the upper (ULA) and lower (LLA) limits of agreement) in five arbitrarily chosen animals in each group. Moreover, we have analyzed the coefficient of variation according to a method described previously (30). The statistical analyses were performed using statistical software SPSS version 26.0 (Statistical Package for Social Science; International Business Machines Corporation, Chicago, IL, USA). Statistical graphs were produced using GraphPad Prism software version 9 (GraphPad Software, Inc., La Jolla, CA, USA). Statistical significance was defined at *p* < 0.05.

## Results

### Cluster analysis

**Figure 2:**
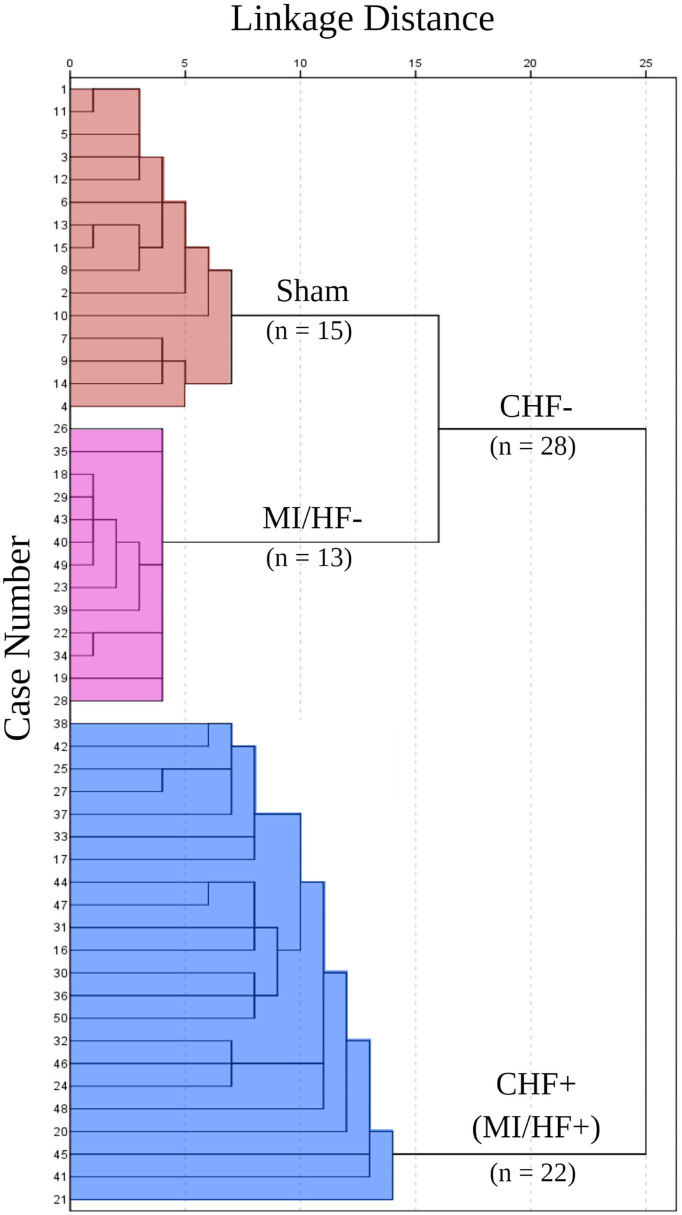
J-tree Cluster analysis. All animals were included in the analysis as cases. Ten echocardiographic variables were used in the cluster analysis: left ventricular internal dimension at end-diastole (LVIDd), left ventricular internal dimension at end-diastole to body weight ratio (LVIDd/BW), left ventricular internal dimension at end-systole (LVIDs), ejection fraction (EF), fractional shortening (FS), left atrium to body weight ratio (LA/BW), left atrium to aorta ratio (LA/Ao), early diastolic transmitral flow velocity (E), early to late diastolic transmitral flow velocities ratio (E/A), and isovolumetric relaxation time (IVRT).

The number of clusters in the data set was determined based on the dendrogram of the J-tree cluster analysis (Figure 2). The animals were joined into two main clusters representing animals with congestive heart failure (CHF+, *n* = 22) and those without congestive heart failure (CHF–, *n* = 28). The CHF+ cluster represented animals with severe anatomical and echocardiographic changes and was named MI with heart failure (MI/HF+, *n* = 22). The CHF– cluster was joined from two clusters. The first one comprises a Sham-operated group (Sham, *n* = 15), while the second one includes the animals with less severe anatomical and echocardiographic changes and was named MI without heart failure (MI/HF–, *n* = 13). On the one hand, the MI/HF+ animals (CHF+ cluster) had large anterolateral transmural MI, hearts were hypertrophied with dilated left ventricles and left atria. Moreover, the heart functions were severely reduced. On the other hand, in the CHF– cluster, MI/HF– animals had medium to large size transmural MI with a slight increase in the heart weight than normal, the LV was dilated, and the LA presented normally to moderate dilation. The cardiac functions were reduced compared to the Sham group but higher than in the MI/HF+ group.

### Conventional echocardiography

The echocardiographic data for assessment of the cardiac structure and function are shown in Table 1 and Supplementary Table S1. Regarding the diastolic function evaluation, the transmitral flow revealed that the E wave was significantly (*p* < 0.0001) elevated, and the A wave was significantly (*p* < 0.001) declined in MI/HF+ group than in the other groups. E/A ratio was significantly (*p* < 0.0001) increased MI/HF+ group than MI/HF– group. There was no significant difference in E, and A waves between MI/HF– and Sham groups, but the E/A ratio was significantly (*p* < 0.05) lower in MI/HF– group than in the Sham group. IVRT was significantly reduced in MI/HF+ and Sham groups compared to the MI/HF– group. The change in the IVRT was not significant between MI/HF+ and Sham groups. Regarding the evaluation of TDI, on the one hand, both septal and lateral s', e' velocities, and e'/a' ratio were significantly lowered in MI/HF+ group compared to the other groups. On the other hand, the septal and lateral a' velocities, the E/septal e', E/lateral e', and E/e' ratios were significantly higher in MI/HF+ group than in the other groups. There were no significant changes in the TDI evaluations between the MI/HF– and the Sham groups. The assessment of the LV structure exhibited a significant increase in LVIDd, LVIDd/BW (*p* < 0.001), LVPWd, and LVIDs (*p* < 0.0001) in MI/HF+ group than in the Sham group. The same parameters were significantly (*p* < 0.05), LVPWd (*p* < 0.01), increased in MI/HF– group than in the Sham group. LVIDd, LVIDd/BW (*p* < 0.05), and LVIDs (*p* < 0.01) were significantly higher in MI/HF+ than MI/HF– groups. Changes in LVPWs were not significant among the groups. There was a significant (*p* < 0.05) increase in the LVM in MI/HF+ and MI/HF– groups compared to the Sham group. The difference in the LVPWd between MI/HF+ and MI/HF– groups was not significant. The IVSd was significantly lower in MI/HF+ group compared to the Sham one. LVM in MI/HF+ animals was significantly higher than that of the other two groups. Evaluation of the LV function revealed a significant (*p* < 0.0001) reduction in both EF and FS in the MI/HF+ group vs. the Sham group and a significant (*p* < 0.01) decrease in the same parameters in the MI/HF– group compared with the Sham group. Despite their reduction, the alteration of EF and FS between MI/HF+ and MI/HF– groups was not significant. There was a significant elevation of LADS, LA/Ao, and LA/BW parameters in the MI/HF+ group than in other groups. Regarding AoDd, it presented a significant elevation in MI/HF– group than other groups. Assessment of the aortic blood flow (LVOT) and the pulmonary blood flow (RVOT) tracts showed that there was a significant reduction in the PV, CO, and VTI parameters of both LVOT and RVOT in the MI/HF+ group than in the other groups. The alterations in these parameters were not significant among MI/HF– and Sham groups.

**Table 1:** Assessment of cardiac structure and function using conventional echocardiography.

**Variables**	**Sham**	**MI/HF−**	**MI/HF+**
	**(*n* = 15)**	**(*n* = 12)**	**(*n* = 23)**
**Diastolic echocardiographic data used for cluster analysis**			
E (cm/s)	82.31 ± 4.01	77.14 ± 2.93	111.15 ± 15.64***^†^^†^^†^^†^
E/A	1.52 ± 0.15	1.14 ± 0.09*	4.55 ± 1.06**^†^^†^^†^^†^
IVRT (ms)	23.80 ± 2.95	34.25 ± 3.02***	22.43 ± 4.57^†^^†^^†^^†^
**Other diastolic echocardiographic data**			
A (cm/s)	54.65 ± 6.24	67.64 ± 3.59	26.13 ± 8.85***^†^^†^^†^^†^
Septal s' (cm/s)	4.25 ± 0.77	3.95 ± 0.68	2.85 ± 0.41****^†^^†^^†^
Septal e' (cm/s)	5.58 ± 1.14	4.77 ± 0.86	4.00 ± 0.57***
Septal a' (cm/s)	4.07 ± 0.95	3.86 ± 0.71	5.27 ± 0.99**^†^^†^^†^
Septal e'/a' (cm/s)	1.39 ± 0.16	1.24 ± 0.17	0.78 ± 0.15****^†^^†^^†^
E/Septal e' (cm/s)	15.36 ± 3.24	16.75 ± 3.53	28.64 ± 7.27****^†^^†^^†^
Lateral s' (cm/s)	5.02 ± 0.61	4.48 ± 0.47	3.85 ± 0.46****^†^
Lateral e' (cm/s)	5.98 ± 0.99	5.12 ± 0.75	4.61 ± 0.66***
Lateral a' (cm/s)	4.97 ± 1.13	4.21 ± 0.69	6.21 ± 0.95*^†^^†^^†^^†^
Lateral e'/a' (cm/s)	1.23 ± 0.18	1.22 ± 0.09	0.76 ± 0.18****^†^^†^^†^^†^
E/Lateral e' (cm/s)	14.14 ± 2.50	15.42 ± 2.67	24.69 ± 5.51****^†^^†^^†^
E/e'	14.75 ± 2.80	16.08 ± 3.04	26.66 ± 6.20****^†^^†^^†^
**Echocardiographic data used for cluster analysis**			
LVIDd (mm)	8.31 ± 0.70	10.59 ± 0.48*	11.46 ± 0.53***^†^
LVIDd/BW (mm/kg)	15.37 ± 2.58	20.86 ± 1.22*	25.76 ± 3.22***^†^
LVIDs (mm)	5.01 ± 0.50	8.02 ± 0.64*	9.45 ± 0.85****^†^^†^
EF (%)	80.10 ± 3.25	59.03 ± 5.68**	46.55 ± 11.06****
FS (%)	41.96 ± 3.38	25.89 ± 3.48**	19.23 ± 5.72****
LA/Ao	1.54 ± 0.19	1.90 ± 0.17*	2.45 ± 0.34****
LA/BW (mm/kg)	9.77 ± 1.24	14.59 ± 0.78*	19.53 ± 2.53****^†^^†^

Data are means ± SD. n, number of animals; Sham, Sham-operated animals; MI/HF–, animals with myocardial infarction and no heart failure; MI/HF+, animals with myocardial infarction and heart failure; BW, body weight; LVIDd, left ventricular internal dimension at end-diastole; LVIDd/BW, left ventricular internal dimension at end-diastole to body weight ratio; LVIDs, left ventricular internal dimension at end-systole; EF, ejection fraction; FS, fractional shortening; LA/Ao, left atrium to aorta ratio; LA/BW, left atrium to body weight ratio; E, early diastolic transmitral flow velocity; A, late diastolic transmitral flow velocity; E/A, early to late diastolic transmitral flow velocities ratio; IVRT, isovolumetric relaxation time; s', left ventricular wall velocity at systole; e', left ventricular wall velocity at early diastole; a', left ventricular wall velocity at late diastole; e'/a', early to late diastolic velocity of the left ventricular wall; E/é, early diastolic velocity mitralis to the early diastolic velocity of the LV wall ratio. *p <0.05, **p <0.01, ***p <0.001, and **** p <0.0001 vs. Sham; ^†^p <0.05, ^†^^†^p <0.01, ^†^^†^^†^p <0.001, and ^†^^†^^†^p <0.0001 vs. MI/HF–.

### Color M-mode echocardiography/intraventricular pressure gradient

**Figure 3:**
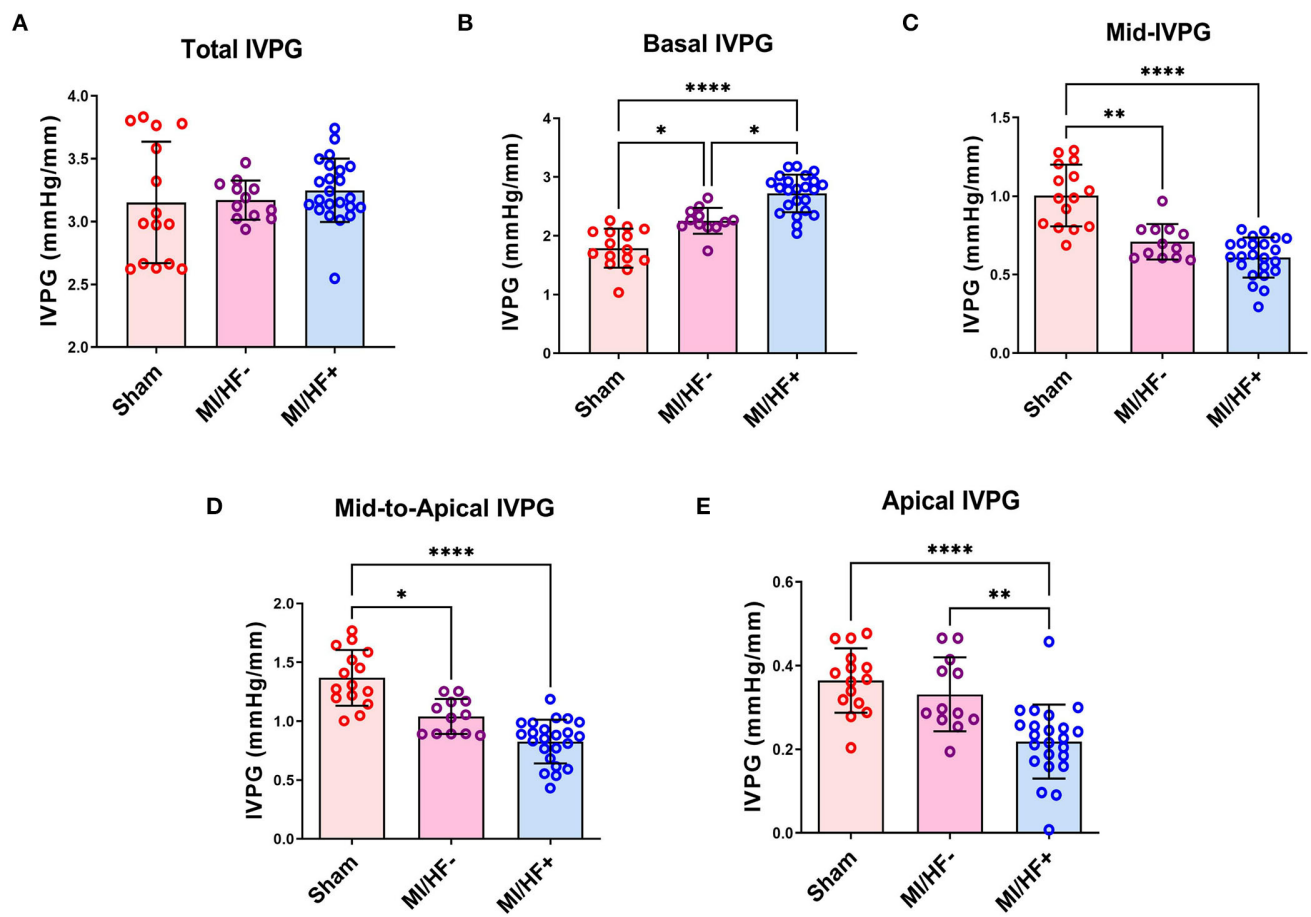
Scatter plots with bars of the IVPG indices in different groups. Data presented as mean ± SD. The total IVPG showed no significant change among different study groups **(A)**. Basal IVPG was significantly increased in MI/HF– and MI/HF+ groups compared to the Sham one **(B)**. The mid-IVPG **(C)** and the mid-to-apical IVPG **(D)** presented a significant decline in MI/HF– and MI/HF+ groups compared to the Sham group. The apical IVPG decreased significantly in the MI/HF+ group compared to the other groups **(E)**. *p < 0.05, **p < 0.01, and ****p < 0.0001.

The CMME assessment of the LV inflow is shown in Figure 3 and Supplementary Table S2. The IVPG was affected by the presence of CHF, the MI/HF+ group presented significantly lower values of various IVPG parameters. There was no significant change in the total IVPG among different groups. Only the basal IVPG showed a significant increase in MI/HF+ group compared to the MI/HF– and Sham groups (2.72 ± 0.31 vs. 2.25 ± 0.22 and 1.79 ± 0.33 mmHg/mm; respectively). Also, it was significantly (*p* < 0.05) increased in MI/HF– group than Sham group. Mid-to-apical IVPG and mid-IVPG were significantly reduced in both MI/HF+ and MI/HF– groups compared to the Sham group (0.83 ± 0.19 and 1.04 ± 0.15 vs. 1.37 ± 0.23 mmHg/mm; for mid-to-apical IVPG and 0.61 ± 0.13 and 0.71 ± 0.11 vs. 1.00 ± 0.19 mmHg/mm; for mid IVPG, respectively). There were no significant changes in these IVPG indices between the MI/HF+ and MI/HF– groups. On the other hand, the apical IVPG showed a significant decrement in MI/HF+ group compared to the other groups. These changes were not significant between the MI/HF– and Sham groups.

### Hemodynamic evaluation of the intraventricular pressure

**Figure 4:**
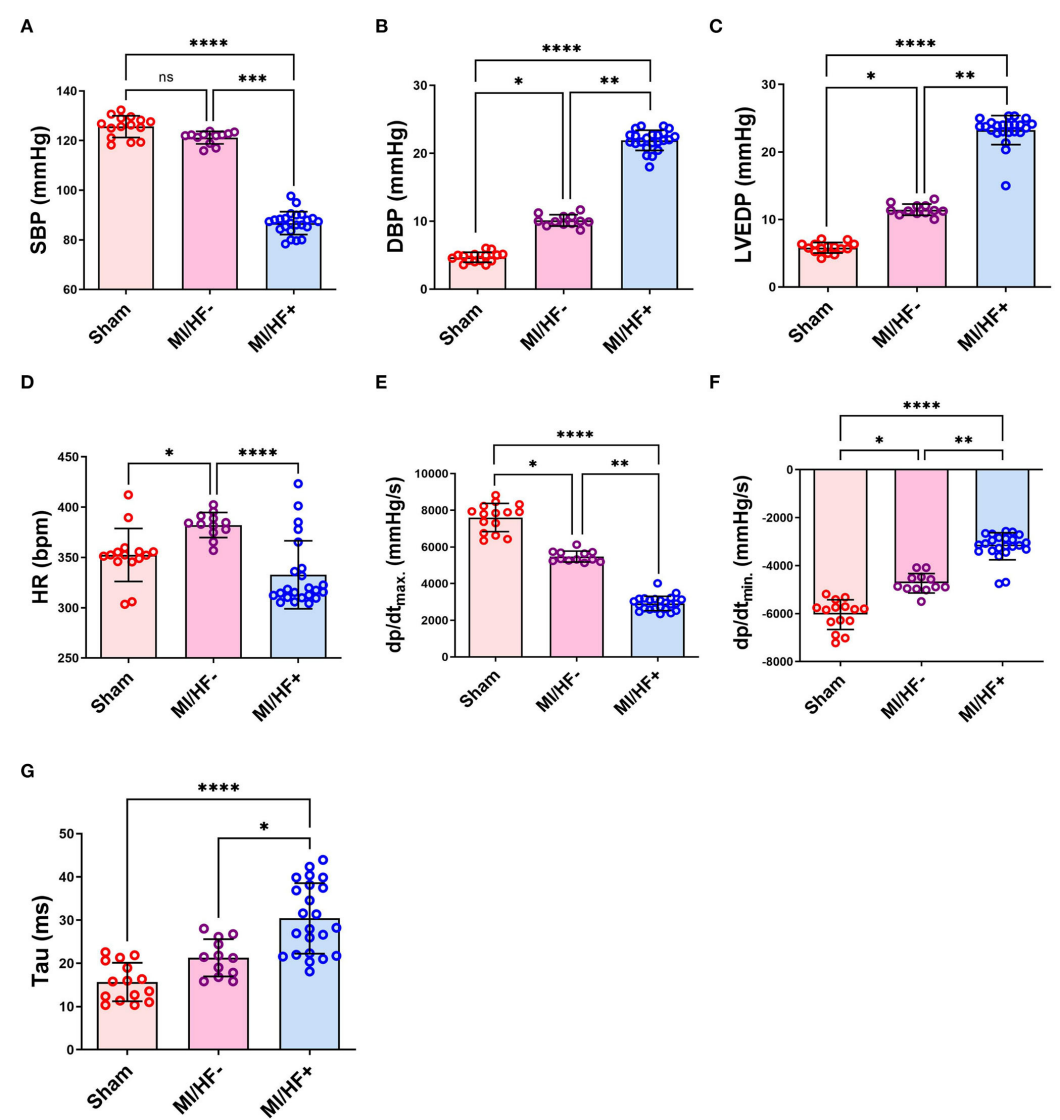
Scatter plots with bars presenting a quantitative comparison of various hemodynamic parameters in different groups including systolic blood pressure (SBP) **(A)**, diastolic blood pressure (DBP) **(B)**, left ventricular end-diastolic pressure (LVEDP) **(C)**, heart rate (HR) **(D)**, positive left ventricular pressure derivative (dP/dt_max._) **(E)**, negative left ventricular pressure derivative (dP/dt_min._) **(F)**, and time constant of isovolumic relaxation (Tau) **(G)**. Data presented as mean ± SD. *p < 0.05, **p < 0.01, ***p < 0.001, and ****p < 0.0001.

The hemodynamic data describing the invasive intraventricular blood pressure (IVP) are illustrated in Figure 4 and Supplementary Table S3. The SBP was significantly lower in MI/HF+ group than in the MI/HF– and Sham groups. It did not significantly change in MI/HF– group compared to the Sham group. On the one hand, load-dependent LV contractility (dP/dt_max._) and relaxation (dP/dt_min._) indices were significantly decreased in MI/HF+ vs. Sham (*p* < 0.0001), MI/HF+ vs. MI/HF– (*p* < 0.01), and MI/HF– vs. Sham (*p* < 0.05). There was a significant decrement in the HR in the MI/HF+ group than MI/HF– group (*p* < 0.0001). The variation in the HR was not significant between MI/HF+ and Sham groups. On the other hand, the HR was significantly (*p* < 0.05) increased in MI/HF– group than in the Sham group.

On the contrary, the DBP and the LVEDP were significantly increased in MI/HF+ group compared to the MI/HF– group (*p* < 0.01) and Sham group (*p* < 0.0001) (21.91 ± 2.62 vs. 10.11 ± 0.83 and 4.70 ± 0.71 mmHg; for DBP and 23.24 ± 2.62 vs. 11.45 ± 0.83 and 5.80 ± 0.76 mmHg; for LVEDP, respectively). Tau (τ) presented a double-fold significant (*p* < 0.0001) increase in MI/HF+ than the Sham group and about 43% significant (*p* < 0.05) increase in MI/HF+ than MI/HF– group. The alteration of Tau was not significant between MI/HF– and Sham groups; however, it was about 36% higher in the MI/HF– group than in the Sham group.

### Anatomical characteristics

**Figure 5:**
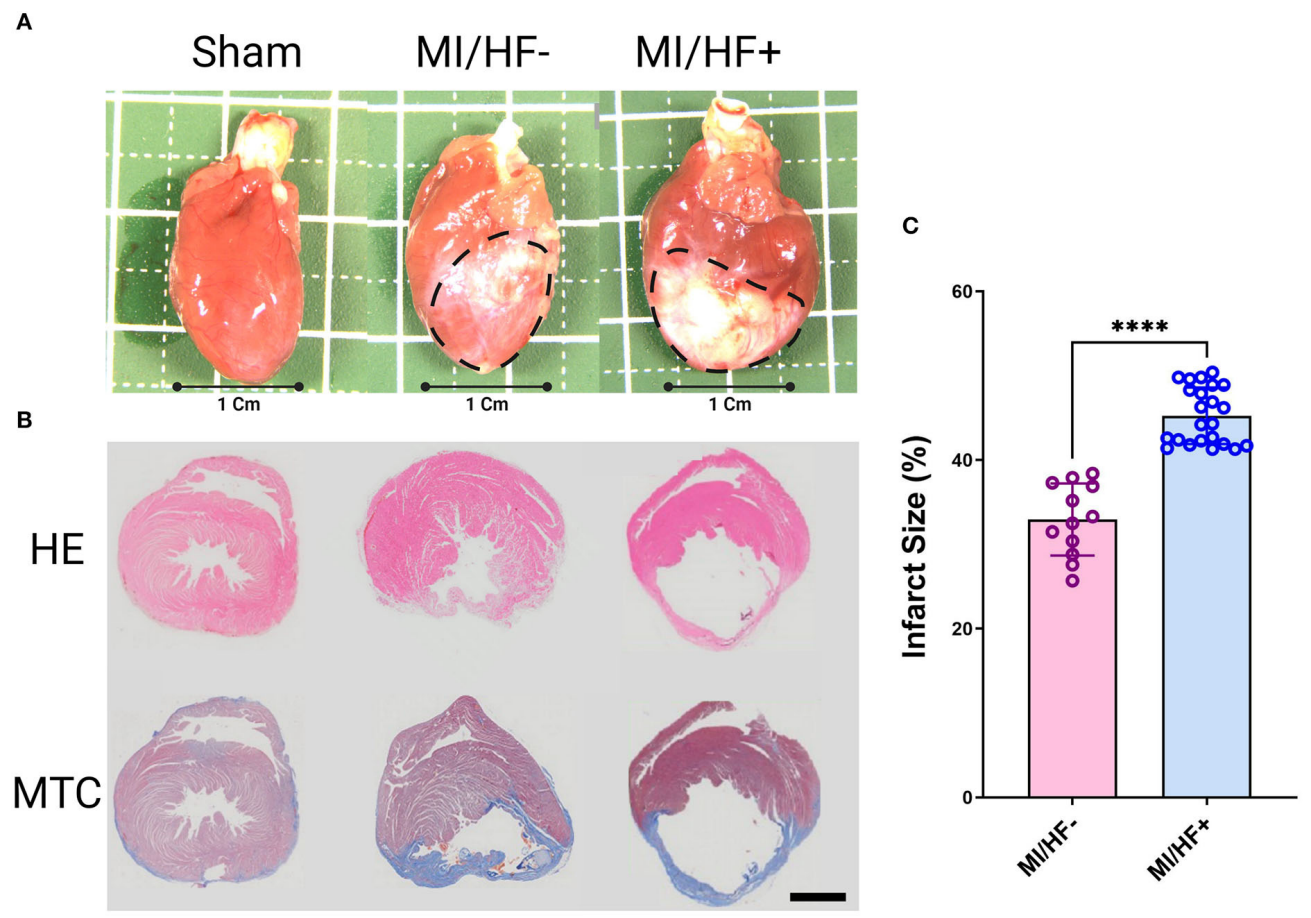
Representative gross photographs **(A)**, Hematoxylin and Eosin (HE), and Masson's trichrome (MTC) stained images of hearts **(B)** 6 months post-surgery. Quantitative assessment of the infarct size % in MI/HF– and MI/HF+ groups **(C)**. The Black dotted area indicated an infarction area. Scale bar: 300 μm. Data presented as mean ± SD. ****p < 0.0001.

The anatomical data are relative to the cluster analysis groups. Anatomical variables are presented in Table 2. The MI/HF+ group showed the most severe anatomical changes with a significant reduction in BW and BW gain (*p* < 0.0001) compared to the other groups that did not present in-between differences. On the other hand, the heart wt.-to-BW ratio and the liver wet-to-dry weight ratio were significantly higher in the MI/HF+ group than in the other groups. The heart size increased in MI groups compared to the Sham group and in MI/HF+ group compared to the MI/HF– group as presented in Figure 5A. The lung wet-to-dry weight ratio was significantly (*p* < 0.05) higher in the MI/HF+ group compared to the Sham group. However, this increase was not significantly different compared to the MI/HF– group. Moreover, the MI/HF– group had a significantly higher heart wt.-to-BW ratio than the Sham group (*p* < 0.05). There were no significant differences between MI/HF– and Sham groups regarding lung and liver wet-to-dry weight ratios.

**Table 2:** Anatomical characteristics of different groups.

**Variables**	**Sham**	**MI/HF−**	**MI/HF+)**
	**(*n* = 15)**	**(*n* = 12)**	**(*n* = 23)**
BW (gm)	549.27 ± 57.95	508.61 ± 22.14	443.39 ± 42.55*^†^^†^
BW Gain (gm)	190.21 ± 54.25	138.93 ± 33.01	88.00 ± 32.53****^†^^†^
Heart Wt./BW (gm/kg)	2.43 ± 0.17	3.76 ± 0.27*	5.58 ± 0.97****^†^^†^
Lung Wet/dry Wt. (gm)	4.29 ± 0.73	4.43 ± 0.36	5.23 ± 1.35*
Liver Wet/dry Wt. (gm)	3.10 ± 0.27	3.14 ± 0.41	3.40 ± 0.29*^†^

Data are means ± SD. n, number of animals. Sham, Sham-operated animals; MI/HF–, animals with myocardial infarction and no heart failure; MI/HF+, animals with myocardial infarction and heart failure; BW, body weight; ^*^p <0.05 and ^****^p <0.0001 vs. Sham; ^†^p <0.05 and ^†^^†^p <0.01 vs. MI/HF–.

### Infarct size %

The infarct size % was significantly (*p* < 0.0001) higher in MI/HF+ group than MI/HF– group (45.26 ± 3.29, 32.96 ± 4.09; respectively) as illustrated in Figures 5B,C.

### Relationship between IVPG and diastolic echocardiographic parameters

Table 3 presents the data for correlation between different IVPG indices and the conventional diastolic echocardiographic parameters. On the one hand, Basal IVPG displayed a significant positive correlation with E, A, and E/A. On the other hand, mid-to-apical IVPG, mid-IVPG, and apical IVPG were significantly negatively correlated with the same measures. Besides, none of the IVPG indices was correlated with the IVRT. Total IVPG was significantly negatively correlated with septal s' and septal e' (*r* = −0.30, *p* = 0.037, and *r* = −0.28, *p* = 0.047; respectively). On the one hand, basal IVPG presented a significant negative correlation with septal s', septal e', septal e'/a', lateral s', lateral e', and lateral e'/a'. On the other hand, a significant positive correlation was detected between the basal IVPG and septal a', E/septal e', lateral a', E/lateral e', and E/e'. There was a significant positive correlation between the mid-to-apical IVPG, mid-IVPG, and apical IVPG indices and the A, septal s', septal e', septal e'/a', lateral s', lateral e', and lateral e'/a'. The relationship between apical IVPG and lateral s' tends to be positively significant (*r* = −0.24, *p* = 0.093). Furthermore, mid-to-apical IVPG, mid-IVPG, and apical IVPG indices presented a significant negative correlation with septal a', E/septal e', lateral a', E/septal e', and E/e'.

**Table 3:** Correlation between IVPG and conventional echocardiographic parameters.

**Variables**	**Total IVPG**		**Basal IVPG**		**Mid-to-apical IVPG**		**Mid- IVPG**		**Apical IVPG**	
	***R***	***p*-value**	***r***	***p*-value**	***r***	***p*-value**	***r***	***p*-value**	***r***	***p*-value**
**Diastolic echocardiographic data used for cluster analysis**										
E (cm/s)	0.19	0.181	0.58	<0.0001	−0.47	0.0006	−0.36	0.009	−0.55	<0.0001
E/A	0.11	0.442	0.63	<0.0001	−0.61	<0.0001	−0.51	0.0001	−0.64	<0.0001
IVRT (ms)	0.09	0.544	−0.07	0.616	0.16	0.262	0.06	0.663	0.32	0.022
**Other diastolic echocardiographic data**										
A (cm/s)	−0.01	0.969	−0.52	0.0001	0.57	<0.0001	0.46	0.0007	0.63	<0.0001
Septal s' (cm/s)	−0.30	0.037	−0.69	<0.0001	0.45	0.001	0.44	0.0015	0.35	0.0139
Septal e' (cm/s)	−0.28	0.047	−0.57	<0.0001	0.41	0.004	0.36	0.009	0.38	0.007
Septal a' (cm/s)	−0.09	0.536	0.39	0.005	−0.51	0.0001	−0.45	0.001	−0.50	0.0003
Septal e'/a' (cm/s)	−0.11	0.459	−0.70	<0.0001	0.68	<0.0001	0.62	<0.0001	0.62	<0.0001
E/Septal e' (cm/s)	0.25	0.082	0.62	<0.0001	−0.47	0.0006	−0.39	0.005	−0.51	0.0002
Lateral s' (cm/s)	−0.21	0.148	−0.64	<0.0001	0.46	0.0007	0.50	0.0001	0.24	0.093
Lateral e' (cm/s)	−0.26	0.064	−0.56	<0.0001	0.39	0.005	0.37	0.009	0.33	0.019
Lateral a' (cm/s)	0.12	0.407	0.46	0.0009	−0.34	0.016	−0.29	0.044	−0.35	0.013
Lateral e'/a' (cm/s)	−0.23	0.114	−0.69	<0.0001	0.52	0.0001	0.47	0.0006	0.48	0.0004
E/Lateral e' (cm/s)	0.23	0.105	0.62	<0.0001	−0.48	0.0004	−0.40	0.004	−0.50	0.0002
E/e'	0.24	0.088	0.63	<0.0001	−0.48	0.0004	−0.40	0.004	−0.51	0.0001
**Echocardiographic data used for cluster analysis**										
LVIDd (mm)	0.08	0.603	0.68	<0.0001	−0.74	<0.0001	−0.73	<0.0001	−0.56	<0.0001
LVIDd/BW (mm/kg)	0.12	0.409	0.69	<0.0001	−0.69	<0.0001	−0.67	<0.0001	−0.53	<0.0001
LVIDs (mm)	0.02	0.869	0.74	<0.0001	−0.80	<0.0001	−0.77	<0.0001	−0.62	<0.0001
EF %	−0.06	0.67	−0.70	<0.0001	0.73	<0.0001	0.70	<0.0001	0.57	<0.0001
FS %	−0.07	0.616	−0.71	<0.0001	0.74	<0.0001	0.72	<0.0001	0.58	<0.0001
LA/AO	0.11	0.441	0.65	<0.0001	−0.63	<0.0001	−0.57	<0.0001	−0.57	<0.0001
LA/BW (mm/kg)	0.13	0.371	0.76	<0.0001	−0.72	<0.0001	−0.69	<0.0001	−0.57	<0.0001

### Relationship between IVPG and other conventional echocardiographic parameters

The data for correlation between different IVPG indices and the other echocardiographic parameters exploited for cluster analysis are presented in Table 3. Basal IVPG displayed a significant positive correlation with LVIDd, LVIDd/BW, LVIDs, LA/Ao, and LA/BW. Mid-to-apical IVPG, mid-IVPG, and apical IVPG were significantly negatively correlated with the same echocardiographic parameters. Moreover, a significant negative correlation was observed between the basal IVPG and EF and FS (*r* = −0.70, *p* < 0.0001, and *r* = −0.71, *p* < 0.0001, respectively). On contrary, a significant positive correlation was observed among mid-to-apical IVPG, mid-IVPG, and apical IVPG with the same parameters (*r* = 0.73, *p* < 0.0001, *r* = 0.70, *p* < 0.0001, and *r* = 0.57, *p* < 0.0001, respectively with EF) and (*r* = 0.74, *p* < 0.0001, *r* = 0.72, *p* < 0.0001, and *r* = 0.58, *p* < 0.0001, respectively with FS). There was no significant correlation between the total IVPG, and these echocardiographic parameters employed for cluster analysis.

### Relationship between IVPG and the hemodynamic (IVP) parameters

The data presenting the relationship between different IVPG indices, and the hemodynamic parameters are presented in Table 4. No significant correlation was detected between the total IVPG and the different hemodynamic indices. Meanwhile, a significant negative correlation was observed between the basal IVPG and the SBP, HR, and dP/dt_max._. Also, basal IVPG was significantly positively correlated with the DBP, LVEDP, dP/dt_min._, and Tau. Mid-to-apical IVPG, mid-IVPG, and apical IVPG indices displayed a significant positive correlation with SBP and dP/dt_max._. Moreover, a significant positive correlation was noticed between the apical IVPG and the HR. Contrariwise, a significant negative correlation was realized among the mid-to-apical IVPG, mid-IVPG, and apical IVPG indices and DBP, LVEDP, dP/dt_min._, and Tau, respectively.

**Table 4:** Correlation between IVPG and hemodynamic (IVP) parameters.

**Variables**	**Total IVPG**		**Basal IVPG**		**Mid-to-apical IVPG**		**Mid- IVPG**		**Apical IVPG**	
	***r***	***p*-value**	***r***	***p*-value**	***r***	***p*-value**	***r***	***p*-value**	***r***	***p*-value**
SBP (mmHg)	−0.13	0.351	−0.73	<0.0001	0.68	<0.0001	0.59	<0.0001	0.68	<0.0001
DBP (mmHg)	0.08	0.579	0.76	<0.0001	−0.76	<0.0001	−0.70	<0.0001	−0.67	<0.0001
LVEDP (mmHg)	0.10	0.505	0.76	<0.0001	−0.76	<0.0001	−0.70	<0.0001	−0.66	<0.0001
HR (bpm)	0.08	0.591	−0.12	0.400	0.20	0.164	0.12	0.412	0.31	0.027
dP/dt_max._ (mmHg/s)	−0.15	0.285	−0.77	<0.0001	0.75	<0.0001	0.70	<0.0001	0.65	<0.0001
dP/dt_min._ (mmHg/s)	−0.02	0.888	0.70	<0.0001	−0.75	<0.0001	−0.71	<0.0001	−0.61	<0.0001
Tau (ms)	0.03	0.827	0.55	<0.0001	−0.62	<0.0001	−0.54	<0.0001	−0.60	<0.0001

### Relationship between IVPG and the anatomic and histologic parameters

Table 5 describes the relationship between the IVPG measures and the anatomic and histologic parameters. There was no observed significant correlation between the total IVPG and various anatomic and histologic parameters. However, a significant negative correlation was observed between the basal IVPG and the BW, and Wt. gain. Meanwhile, the correlation between the basal IVPG and the heart wt./BW, lung wet-to-dry weight ratio, and the infarct size was significantly positive. There was a significant positive correlation among the mid-to-apical IVPG, mid-IVPG, and apical IVPG indices and the BW, and wt. gain. These IVPG indices also presented a significant negative correlation with the heart weight-to-BW ratio and the infarct size. Likewise, a significant negative correlation between the lung wet-to-dry weight ratio and the mid-to-apical and apical IVPG measures (*r* = −0.33, *p* = 0.0201, and *r* = −0.39, *p* = 0.005, respectively) was noticed, but correlation with mid-IVPG was not. None of the IVPG indices showed a significant correlation with the liver wet-to-dry weight ratio.

**Table 5:** Correlation between IVPG, anatomic parameters, and infarct size.

**Variables**	**Total IVPG**		**Basal IVPG**		**Mid-to-apical IVPG**		**Mid- IVPG**		**Apical IVPG**	
	***r***	***p*-value**	***r***	***p*-value**	***r***	***p*-value**	***r***	***p*-value**	***r***	***p*-value**
BW (gm)	0.03	0.840	−0.53	<0.0001	0.61	<0.0001	0.61	<0001	0.45	0.001
Wt. gain (gm)	0.11	0.468	−0.50	0.0002	0.67	<0.0001	0.65	<0.0001	0.50	0.0002
Heart Wt./BW (gm/kg)	0.16	0.262	0.75	<0.0001	−0.69	<0.0001	−0.66	<0.0001	−0.56	<0.0001
Lung Wt. wet/dry (gm)	−0.07	0.644	0.30	0.033	−0.33	0.0201	−0.25	0.075	−0.39	0.005
Liver Wt. wet/dry (gm)	−0.08	0.594	0.18	0.203	−0.24	0.092	−0.21	0.139	−0.23	0.106
Infarct size (%)	0.08	0.581	0.75	<0.0001	−0.79	<0.0001	−0.77	<0.0001	−0.60	<0.0001

### Multivariate regression analysis

The multivariable analyses are shown in Table 6. Total IVPG was significantly correlated with LVIDd, LVIDs, EF, FS, DBP, LVEDP, BW, weight gain, and infarct size. Basal IVPG, mid-to-apical IVPG, mid-IVPG, and apical IVPG indices were significant predictors of the chronic CHF following MI. According to the multivariate analysis, they were significantly correlated with all echocardiographic parameters employed for the cluster analysis except IVRT, as well as all hemodynamic parameters except the HR where only apical IVPG was a significant predictor (*R*^2^ = 0.098, *p* = 0.027). Moreover, they were significantly correlated with the other echocardiographic parameters, BW, Wt. gain, and Heart wt./BW. Lung wet/dry Wt. ratio was significantly affecting mid-to-apical and apical IVPGs (*R*^2^ =0.108, *p* = 0.020; *R*^2^ = 0.150, *p* = 0.005, respectively).

**Table 6:** Multivariable associations between IVPG indices, conventional echocardiographic, hemodynamic, anatomical, and histologic parameters.

**Variables**	**Total IVPG**		**Basal IVPG**		**Mid-to-apical IVPG**		**Mid- IVPG**		**Apical IVPG**	
	***R* ^2^**	***p*-value**	***R* ^2^**	***p*-value**	***R* ^2^**	***p*-value**	***R* ^2^**	***p*-value**	***R* ^2^**	***p*-value**
**Diastolic echocardiographic data used for cluster analysis**										
E (cm/s)	0.037	0.707	0.331	0.022	0.220	0.0006	0.132	0.009	0.306	<0.0001
E/A	0.012	0.246	0.396	0.018	0.370	<0.0001	0.259	0.0002	0.410	<0.0001
IVRT (ms)	0.008	0.474	0.005	0.840	0.026	0.262	0.004	0.663	0.104	0.023
**Other diastolic echocardiographic data**										
Septal s' (cm/s)	0.088	0.865	0.481	0.0003	0.203	0.001	0.192	0.002	0.120	0.014
Septal e' (cm/s)	0.080	0.849	0.324	0.01	0.164	0.004	0.131	0.01	0.144	0.007
Septal a' (cm/s)	0.008	0.068	0.150	0.177	0.264	0.0001	0.204	0.001	0.246	0.0003
Septal e'/a' (cm/s)	0.011	0.142	0.484	0.006	0.467	<0.0001	0.383	<0.0001	0.386	<0.0001
E/Septal e' (cm/s)	0.062	0.911	0.379	0.007	0.221	0.0006	0.150	0.006	0.257	0.0002
Lateral s' (cm/s)	0.043	0.617	0.405	0.004	0.214	0.0007	0.254	0.0002	0.058	0.093
Lateral e' (cm/s)	0.069	0.893	0.312	0.007	0.153	0.005	0.134	0.009	0.110	0.019
Lateral a' (cm/s)	0.014	0.799	0.209	0.024	0.114	0.016	0.082	0.044	0.121	0.013
Lateral e'/a' (cm/s)	0.051	0.736	0.471	0.001	0.272	0.0001	0.219	0.0006	0.233	0.0004
E/Lateral e' (cm/s)	0.054	0.790	0.390	0.007	0.231	0.0004	0.163	0.004	0.253	0.0002
E/e'	0.060	0.856	0.393	0.007	0.231	0.0004	0.159	0.004	0.261	0.0001
A (cm/s)	0.000	0.113	0.271	0.099	0.323	<0.0001	0.213	0.0008	0.394	<0.0001
**Echocardiographic data used for cluster analysis**										
LVIDd (mm)	0.006	0.043	0.464	0.021	0.555	<0.0001	0.532	<0.0001	0.315	<0.0001
LVIDd/BW (mm/kg)	0.014	0.128	0.482	0.007	0.475	<0.0001	0.447	<0.0001	0.283	<0.0001
LVIDs (mm)	0.001	0.014	0.545	0.005	0.632	<0.0001	0.591	<0.0001	0.383	<0.0001
EF %	0.004	0.047	0.486	0.007	0.531	<0.0001	0.493	<0.0001	0.329	<0.0001
FS %	0.005	0.046	0.506	0.006	0.553	<0.0001	0.517	<0.0001	0.335	<0.0001
LA/AO	0.012	0.146	0.427	0.017	0.392	<0.0001	0.322	<0.0001	0.322	<0.0001
LA/BW (mm/kg)	0.017	0.112	0.578	0.002	0.512	<0.0001	0.474	<0.0001	0.320	<0.0001
**Hemodynamic parameters**										
SBP (mmHg)	0.018	0.179	0.527	0.004	0.467	<0.0001	0.351	<0.0001	0.459	<0.0001
DBP (mmHg)	0.006	0.049	0.571	0.005	0.582	<0.0001	0.495	<0.0001	0.444	<0.0001
LVEDP (mmHg)	0.009	0.062	0.584	0.004	0.574	<0.0001	0.487	<0.0001	0.440	<0.0001
Heart rate (bpm)	0.006	0.265	0.015	0.699	0.040	0.164	0.014	0.412	0.098	0.027
dP/dt_max._ (mmHg/s)	0.024	0.130	0.593	0.003	0.566	<0.0001	0.485	<0.0001	0.425	<0.0001
dP/dt_min._ (mmHg/s)	0.000	0.008	0.487	0.016	0.557	<0.0001	0.502	<0.0001	0.371	<0.0001
Tau (ms)	0.001	0.095	0.307	0.049	0.380	<0.0001	0.293	<0.0001	0.354	<0.0001
**Anatomic parameters**										
BW (gm)	0.001	0.033	0.281	0.099	0.375	<0.0001	0.366	<0.0001	0.203	0.001
BW gain (gm)	0.011	0.005	0.250	0.221	0.443	<0.0001	0.424	<0.0001	0.252	0.0002
Heart Wt./BW (gm/kg)	0.026	0.199	0.563	0.002	0.483	<0.0001	0.442	<0.0001	0.309	<0.0001
Lung Wt. wet/dry (gm)	0.004	0.155	0.092	0.273	0.108	0.020	0.065	0.075	0.150	0.005
Liver Wt. wet/dry (gm)	0.006	0.129	0.034	0.830	0.058	0.092	0.045	0.139	0.053	0.106
**Histologic parameter**										
Infarct size (%)	0.006	0.032	0.567	0.004	0.624	<0.0001	0.593	<0.0001	0.363	<0.0001

### Intra-observer variability to assess the reproducibility

Analysis of the intra-observer variability using the Bland–Altman test for different IVPG indices in various groups of the current study is illustrated in Table 7. Segmental IVPG indices and sham and MI/HF– groups tended to present higher CV (%). However, in most of them, it is lower than 15%. Hence, the intra-observer agreements were assumed to be reasonable.

**Table 7:** Intra-observer reproducibility of the IVPG variables.

**Variables**	**Bias**	**ULA**	**LLA**	**CV (%)**
**Total IVPG**
Sham group	−0.00966	0.2652	−0.2846	5.331
MI/HF– group	−0.03148	0.274	−0.337	7.519
MI/HF+ group	−0.0023	0.2103	−0.2149	8.149
**Basal IVPG**
Sham group	−0.0034	0.1146	−0.1214	23.99
MI/HF– group	0.02456	0.1487	−0.09959	13.92
MI/HF+ group	0.01476	0.1796	−0.1501	13.5
**Mid-to-apical IVPG**
Sham group	−0.01156	0.08824	−0.1114	15.78
MI/HF– group	0.02657	0.1486	−0.09547	15.77
MI/HF+ group	−0.02074	0.1537	−0.1952	6.536
**Mid-IVPG**
Sham group	0.002004	0.1622	−0.1582	18.29
MI/HF– group	−0.01594	0.06701	−0.09889	12.32
MI/HF+ group	0.03007	0.06919	−0.00904	9.857
**Apical IVPG**
Sham group	−0.01927	0.07533	−0.1139	5.978
MI/HF– group	0.02074	0.09358	−0.05209	19.7
MI/HF+ group	0.00992	0.05357	−0.03373	12.08

*ULA, 95% upper limit of agreement; LLA, 95% lower limit of agreement; CV, coefficient of variation*.

## Discussion

To induce LV dysfunction and subsequent HF, MI rats are among the most used models. The time that elapses to develop HF is often long. However, this period following MI is variable, where the development of CHF is limited to animals with moderate to large size infarctions. Meanwhile, those with small or sometimes medium size infarctions exhibit only LV dysfunction with no indications of CHF (1, 4, 31). The identification of CHF progression in MI rats is of great importance, especially during the assessment of the therapeutic protocols for CHF. Moreover, the precise diagnosis of CHF without invasive techniques such as catheter-based hemodynamics and postmortem examinations was difficult to attain. Hence, there is an increasing interest in an accurate noninvasive method to diagnose CHF following long-term MI rats (4). Over the years, massive research endeavors have focused on the diagnosis of CHF in MI animal models. Baily et al. (32) could evaluate the cardiac changes and LV dysfunction following MI using echocardiography for the first time in 1993 and followed by Litwin et al. (33) who could 1994 assess the cardiac structural and functional alterations due to LV remodeling after MI in rats. Later in 2000, the J-tree cluster analysis was used for the first time by Sjaastad et al. (5) to separate the rats that developed CHF after MI from those without CHF depending on certain echocardiographic and anatomic parameters. After that, Prunier et al. (34) could conduct the first combined analysis of the mitral inflow and mitral annular velocity to evaluate the rat LVEDP in 2002. In this study, based on certain echocardiographic measures in the cluster analysis, we could discriminate the animals into three different groups are Sham, MI/HF–, and MI/HF+. MI/HF+ presented the most severe echocardiographic, hemodynamic, and anatomic alterations compared to the other groups. Moreover, they exhibited larger infarct size than MI/HF– group. These findings come in line with those from previous studies (4, 5, 35) declared that large size MI due to high site occlusion of LAD in rats could trigger significant impairment of the LV functions. In our study, we have selected male rats as a successful CHF model following MI. A recent investigation declared that there is a distinctive sex variation in the manifestations of CHF after ischemia induction, where female rats can withstand ischemia-induced CHF more than males with the same infarct size and similar reduced EF% (36). Conventional echocardiography revealed that MI/HF+ group presented the most severe cardiac structural alterations in the LVIDd, LVIDd corrected by the BW, LVIDs, LVPWd, LVM, LADs, AoDd, LA/Ao, and LA corrected by the BW. Also, MI/HF+ animals exhibited the most intense cardiac functional deterioration during the systole with drastically reduced EF and FS and sharp changes in the diastolic functional parameters. Likewise, the hemodynamic measurements revealed that MI/HF+ rats had lower SBP, HR, dP/dt_max._, and dP/dt_min._ and increased DBP, LVEDP, and Tau. Consistent with these echocardiographic and hemodynamic results, the anatomic alterations showed elevated heart weight corrected by the body weight and lung and liver weight/dry weights. Furthermore, the assessment of the infarct size where MI/HF+ animals presented the largest infarct zone among the other MI/HF– animals. These findings come in line with previous studies (1, 4, 37, 38). To settle the diagnosis of CHF in an animal model, echocardiographic and hemodynamic criteria are crucial (22, 39). In our study, on the one hand, animals in the CHF+ cluster presented the CHF indicative echocardiographic variations. Moreover, they had LVEDP ≥ 15 mmHg. On the other hand, the CHF– animal cluster did not exhibit failing findings. In addition to the echocardiographic findings, the LVEDP is an essential criterion indicative of CHF that was extensively used previously to completely separate the MI clusters (5). The value of LVEDP can be affected by various factors including position and quality of the catheter, as well as the depth of anesthesia and respiration. However, the substantial elevation of the LVEDP is often correlated with pathological conditions. Moreover, the increment of the LVEDP ≥ 15 mmHg is interpreted as pathological and is indicative of CHF (1, 36). In the current study, all animals in the CHF+ cluster had LVEDP ≥ 15 mmHg. Meanwhile, the LVEDP value in CHF– cluster was lower than 15 mmHg. These findings imply that LVEDP is a valid criterion indicative of CHF even in the absence of echocardiography (5). Being an invasive technique with a possibility to damage the aortic valve and impair the heart function during the recording. Additionally, this approach requires euthanization of the animals. Hence, the employment of LVEDP as a main indicator of CHF becomes limited (40). Hence, echocardiography, which is the gold standard approach to assessing cardiac dysfunctions, provides a substantial noninvasive alternative approach to diagnosing CHF. However, the modest sensitivity and the technical limitations are the main drawbacks of conventional 2D and M-mode echocardiographic measurements (41, 42). Recently, IVPG is an emerging noninvasive, reliable, and highly sensitive preload-independent diastolic function parameter to assess cardiac function, especially during cardiomyopathy (12, 13, 19, 30). Herein, in this study, we report for the first time the effectiveness of IVPG as a novel promising tool for the evaluation of post-infarction chronic CHF. Different conventional echocardiographic measurements for assessment of the diastolic function of rats in our study as Doppler inflow during early diastole (E) in addition to E/A, and TDI indices, particularly septal and lateral e**'** and a**'** indicated that the MI groups, particularly MI/HF+ group, presented abnormal cardiac relaxation during diastole owed to the reduce myocardial compliance. The large infarct zone with severe fibrosis in the MI/HF+ group resulted in an evident deterioration of the diastolic function in these animals. On the other hand, with the worsening of the diastolic function, the systolic functions were also highly affected in MI groups with special reference to the MI/HF+ group, where the EF, FS, LVOT, and RVOT indices (PV, CO, VTI) were highly significantly declined. Many studies have evaluated the systolic and diastolic function of CHF rats following MI (1, 4, 37, 38). However, this assessment using IVPG is not yet investigated. Hence, one of the main goals of this study is to explore the feasibility of IVPG as a diastolic assessment parameter and how this can be evaluated in an animal model with diastolic and systolic dysfunction. Although the total IVPG did not differ among the study groups, segmental IVPG did. Hence, the role of each segment in the diastolic function is worthy to be considered. In this investigation, the mid-IVPG, mid-to-apical IVPG, and apical IVPG indices declined in the MI groups (MI/HF+ and MI/HF–) compared to the other Sham group. Steine et al. (43) declared that the impedance of the apical filling during ischemic LV failure was correlated with the evident decline of the basal-to-apical IVPGs. Moreover, the strong negative correlation between these IVPG measures and the Tau in our study implies that the reduced IVPG measures are owed to the impaired LV relaxation. Furthermore, the cardiac fibrosis (infarct size %) that was highly negatively correlated with these IVPG indices in our study was the main reason for the diminished compliance which comes in line with (44). On contrary, Iwano and his group (45) reported that the elevated LA pressure in patients with diastolic heart failure could preserve the basal IVPD that consequently maintains the early diastolic LV filling even with the diminished LV suction. Our data showed that the basal IVPG increased in MI groups (MI/HF+ and MI/HF–) compared to the sham group with the CHF+ animals presented the greatest value of basal IVPG over all other animals in this study. Moreover, the MI/HF+ group presented abnormally high E and E/e' that were also highly positively correlated with the basal IVPG and accompanied by increased LA pressure. Hence, they are speculated to present LV diastolic dysfunction. In our study, despite the reduced LV suction reflected with the reduced mid-, mid-to-apical, and apical IVPG indices, the total IVPG was preserved in CHF+ animals. This was due to the substantial increment of the basal IVPG from the LA to the mid LV attributed to the elevation of the LA pressure. This high basal IVPG sustains the early diastolic filling (45, 46). In other words, the total IVPG is affected by the LA pressure, thus it is not appropriate for evaluating diastolic function. This finding comes in line with a previous study presented a positive correlation between the pulmonary capillary wedge pressure and the IVPG during exercise, indicating that the IVPG was influenced by LA pressure (27). In our previous study, the total IVPG, which was not significantly changed after administration of milrinone that improved the cardiac contractility, was significantly elevated after administration of the hydroxyethyl starch that increased the preload in a rat model under diverse loading states (13). Compression of the intramyocardial and extracellular elastic constituents during systole results in storage of some of the contraction's energy. LV suction is a result of these components' subsequent elastic recoil (47). Hence, the IVPD is linked to LV systolic and diastolic functions (45). Previous investigations by Courtois et al. (27, 48) and Firstenberg et al. (49) exhibited a relationship between the IVPG and the systolic dysfunction. They speculated that there is an association between the IVPG and the elastic recoil of the LV. Moreover, they provided a mechanism to sustain the filling of the LV at declined diastolic pressure. During diastole, they declared that the impairment of the systolic function results in less energy release with subsequent declined or abnormal intraventricular blood flow. This is in a line with our results.

## Conclusion

Conclusively, compared to the combinatorial assessment of heart structural, and LV systolic and diastolic functional echocardiographic indices, IVPG derived from CMME could offer a promising noninvasive means to diagnose CHF after long-term MI.

## Data availability statement

The raw data supporting the conclusions of this article will be made available by the authors, without undue reservation.

## Ethics statement

The animal study was reviewed and approved by the Experimental Animal Committee of Tokyo University of Agriculture and Technology (Approval No. R02-112) and was conducted following the Guide for the Care and Use of Laboratory Animals at the Tokyo University of Agriculture and Technology.

## Author contributions

HME-H and RT conceived and designed the experiment. HME-H conducted the experiments. HME-H and DM analyzed the data. HME-H, EAM, and RT validated and interpreted the results. HME-H and EAM wrote and drafted the manuscript and wrote the final manuscript. HME-H, EAM, LH, and RT revised and edited the manuscript. All authors have read and agreed to the published version of the manuscript.

## Funding

The research was supported by a full scholarship of the Egypt-Japan Education Partnership-Call 4 (EJEP-4) from the Ministry of Higher Education of the Arab Republic of Egypt.

## Conflict of interest

The authors declare that the research was conducted in the absence of any commercial or financial relationships that could be construed as a potential conflict of interest.

## Publisher's note

All claims expressed in this article are solely those of the authors and do not necessarily represent those of their affiliated organizations, or those of the publisher, the editors and the reviewers. Any product that may be evaluated in this article, or claim that may be made by its manufacturer, is not guaranteed or endorsed by the publisher.
