# Supplementary material for: Intraventricular pressure gradient: A novel tool to assess the post-infarction chronic congestive heart failure
Source: Front Cardiovasc Med. 2022 Aug 16;9:944171. doi: 10.3389/fcvm.2022.944171 (PMC9425054; doi:10.3389/fcvm.2022.944171)
Supplement: Supplementary file 1 [file Data_Sheet_1.docx]

**Supplementary Tables:**

**Table S1:** Assessment of cardiac structure and function using conventional echocardiography.

| **Variables** | | **Sham (n=15)** | **MI/HF- (n=12)** | **MI/HF+ (n=23)** |
| --- | --- | --- | --- | --- |
| **LVPWd (mm)** | | 1.74 ± 0.17 | 2.12 ± 0.15 ** | 2.38 ± 0.28 **** |
| **LVPWs (mm)** | | 2.93 ± 0.35 | 2.73 ± 0.14 | 2.71 ± 0.19 |
| **IVSd (mm)** | | 1.85 ± 0.24 | 1.69 ± 0.18 | 1.63 ± 0.19 * |
| **LVM (mg)** | | 925.32 ± 147.19 | 1504.75 ± 140.06 * | 1829.87 ± 198.87 ****† |
| **LADs (mm)** | | 5.31 ± 0.42 | 7.41 ± 0.25 * | 8.57 ± 0.62 ****† |
| **AoDd (mm)** | | 3.51 ± 0.49 | 3.91 ± 0.29 * | 3.51 ± 0.44 † |
| **LVOT** | **PV (cm/s)** | 103.56 ± 2.34 | 105.76 ± 1.80 | 76.95 ± 7.21***†††† |
|  | **CO (L/m)** | 0.11 ± 0.02 | 0.11 ± 0.01 | 0.07 ± 0.02 ****†††† |
|  | **VTI (cm)** | 5.53 ± 0.23 | 5.48 ± 0.37 | 3.97 ± 0.49 ****†††† |
| **RVOT** | **PV (cm/s)** | 76.25 ± 2.16 | 74.71 ± 2.57 | 56.12 ± 4.45 ****†††† |
|  | **CO (L/m)** | 0.11 ± 0.01 | 0.10 ± 0.01 | 0.07 ± 0.0 ****†††† |
|  | **VTI (cm)** | 5.23 ± 0.23 | 5.17 ± 0.26 | 3.80 ± 0.56 ****†††† |

Data are means ± SD. n, number of animals; Sham, Sham-operated animals; MI/HF-, animals with myocardial infarction and no heart failure; MI/HF+, animals with myocardial infarction and heart failure; LVPWd, left ventricular posterior wall thickness at end-diastole; LVPWs, left ventricular posterior wall thickness at end-systole; LVM, left ventricular mass; LADs, left atrium dimension at systole; AoDd, aortic diameter at diastole; PV, peak velocity; CO, cardiac output; VTI, velocity time integral; LVOT, left ventricular outflow tract; RVOT, right ventricular outflow tract; s’. *P<0.05, **P<0.01, ***P<0.001, and ****P<0.0001 vs. Sham; †P<0.05, ††P<0.01, †††P<0.001, and ††††P<0.0001 vs. MI/HF-.

**Table S2:** Color M-mode echocardiography (CMME) derived intraventricular pressure gradients (IVPG) in different groups.

| **Variable** | **Sham (n=15)** | **MI/HF- (n=12)** | **MI/HF+ (n=23)** |
| --- | --- | --- | --- |
| **Total IVPG (mmHg/mm)** | 3.15 ± 0.48 | 3.17 ± 0.16 | 3.25 ± 0.25 |
| **Basal IVPG (mmHg/mm)** | 1.79 ± 0.33 | 2.25 ± 0.22 * | 2.72 ± 0.31 ****† |
| **Mid-to-apical IVPG (mmHg/mm)** | 1.37 ± 0.23 | 1.04 ± 0.15 * | 0.83 ± 0.19 **** |
| **Mid IVPG (mmHg/mm)** | 1.00 ± 0.19 | 0.71 ± 0.11** | 0.61 ± 0.13 **** |
| **Apical IVPG (mmHg/mm)** | 0.36 ± 0.07 | 0.33 ± 0.09 | 0.22 ± 0.09 ****†† |

Data are means ± SD. IVPG, intraventricular pressure gradient; Mid-to-apical IVPG middle-to-apical intraventricular pressure gradient; Mid IVPG, middle intraventricular pressure gradient. *P<0.05, **P<0.01, and ****P<0.0001 vs. sham; †P<0.05, and ††P<0.01, vs. MI/HF-.

**Table S3:** Hemodynamic assessment in different groups.

| **Variable** | **Sham (n=15)** | **MI/HF- (n=12)** | **MI/HF+ (n=23)** |
| --- | --- | --- | --- |
| **SBP (mmHg)** | 125.62 ± 4.21 | 121.19 ± 2.54 | 86.74 ± 8.02 ****††† |
| **DBP (mmHg)** | 4.70 ± 0.71 | 10.11 ± 0.83 * | 21.91 ± 2.62 ****†† |
| **LVEDP (mmHg)** | 5.80 ± 0.76 | 11.45 ± 0.83 * | 23.24 ± 2.62 ****†† |
| **HR (bpm)** | 352.47 ± 25.44 | 382.25 ± 12.46 * | 332.79 ± 33.78†††† |
| **dP/dt_max._ (mmHg/s)** | 7602.15 ± 745.27 | 5474.59 ± 300.77 * | 2916.06 ± 632.29 ****†† |
| **dP/dt_min._ (mmHg/s)** | -6042.72 ± 598.42 | -4735.99 ± 406.29 * | -3194.16 ± 563.34 ****†† |
| **Tau (**τ) **(ms)** | 15.67 ± 4.28 | 21.27 ± 4.32 | 30.40 ± 8.15 ****† |

Data are means ± SD. SBP, LV systolic blood pressure; DBP, LV diastolic blood pressure; LVEDP, left ventricular end diastolic pressure; HR, heart rate; dP/dt_max_., maximal value of the first derivative of the LV pressure; -dP/dt_min_., minimal value of the first derivative of the LV pressure; Tau (τ), time constant of isovolumic LV relaxation. *P<0.05, and ****P<0.0001 vs. sham; †P<0.05, ††P<0.01, †††P<0.001 and ††††P<0.0001 vs. MI/HF-.
